# *Crataerina melbae* louse flies are competent vectors of *Trypanosoma* sp. associated with nestling mortality in Alpine swifts (*Tachymarptis melba*)

**DOI:** 10.1016/j.ijppaw.2025.101170

**Published:** 2025-11-27

**Authors:** Gastón Moré, Pia Cigler, Pierre Bize, Andrew Hemphill, Saskia A. Keller, Caroline F. Frey, Walter Basso

**Affiliations:** a Institute of Parasitology, Department of Infectious Diseases and Pathobiology, Vetsuisse Faculty, University of Bern, Länggassstrasse 122, 3012, Bern, Switzerland; b National Scientific and Technical Research Council (CONICET), Buenos Aires, Argentina; c Institute for Fish and Wildlife Health, Department of Infectious Diseases and Pathobiology, Vetsuisse Faculty, University of Bern, Länggassstrasse 122, 3012, Bern, Switzerland; d Swiss Ornithological Institute, Seerose 1, 6204, Sempach, Switzerland; e Department of Ecology and Evolution, Biophore, University of Lausanne, CH-1015, Lausanne, Switzerland

**Keywords:** Trypanosomiasis, Louse flies, PCR-sequencing, Switzerland, Alpine swift

## Abstract

Hippoboscidae diptera or louse flies are widely distributed blood-feeding ectoparasites that can transmit blood parasites to their host. Four species are recognized within the *Crataerina* genus, which are parasites of the bird orders Apodiformes and Passeriformes. Alpine swifts (*Tachymarptis melba*) are frequently parasitized by *Crataerina* louse flies during the nesting season. In Switzerland, an increase in Alpine swift nestling mortality has been associated with a *Trypanosoma* sp. infection. In this study, fixed and native specimens of *Craterina* louse-flies collected from Swiss Alpine swifts were analysed to reveal the species identity based on morphotypes and PCR-sequencing, and examined for the presence of *Trypanosoma* sp. using microscopy and PCR. Morphologically, all specimens belonged to the genus *Crataerina*. Based on wing types, both *C. melbae* and *C. acutipennis,* as well as mixed morphotypes, were recorded. Sequencing based on the cytochrome-oxidase 1 (*COI*) gene of 24 flies confirmed the presence of previously defined haplotypes of *C. melbae* and suggest that the morphotypes *C. melbae* and *C. acutipennis* likely constitute a single species. One louse-fly specimen was processed by scanning electron microscopy and elongated organisms resembling trypanosomes were observed on the ventral abdominal surface. Dissected intestines and abdominal surface swabs resulted positive by *Trypanosoma* sp. PCR. Microscopy of native specimens evidenced motile and actively multiplying trypanosomes in the midgut and rectum. All *Trypanosoma* sp. sequences from louse flies were identical to those obtained from tissues of infected Alpine swifts. This study suggests that *C. melbae* is a single species infecting Alpine swifts, regardless of the wing morphotype or sequence type, and that *C. melbae* acts as a competent vector for *Trypanosoma* sp. associated with nestling mortality. Further studies should be focused on environmentally sustainable ways to control *C. melbae*, with the goal to minimize transmission and the impact of trypanosomiasis in Alpine swift populations.

## Highlights

• Different wing morphotypes and *COI* types of *Crataerina melbae* were observed.
• All six different *C. melbae COI* type sequences code for an identical protein.
• *C. melbae* of different morphotypes and *COI* haplotypes all harbored trypanosomes.
• Active, multiplying trypanosomes were detected in *C. melbae* midgut and rectum.
• *C. melbae* are competent vectors of *Trypanosoma* sp. affecting Alpine swifts.

## Graphical abstract

**Figure:**
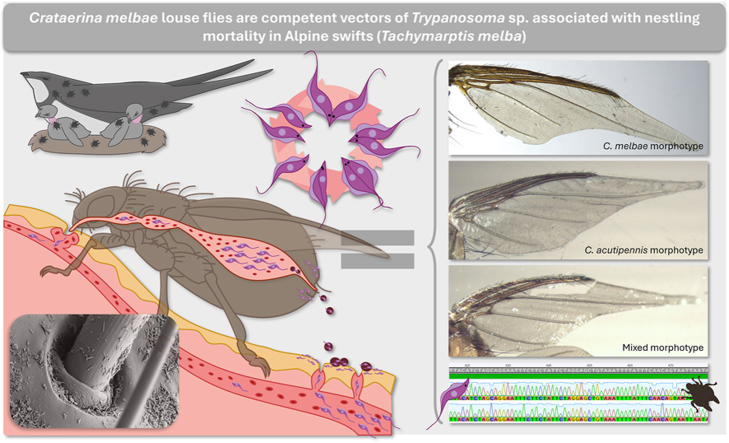

## 1 Introduction

Louse-flies or keds are obligatory dipteran blood sucking ectoparasites from the family Hipposboscidae that can act as vectors of haemoparasites (Santolíková et al., 2022). Several genera of louse flies have been described as parasites of birds, showing different degrees of host specificity (Lehikoinen et al., 2021; Santolíková et al., 2022). Recently, the taxonomic keys for European Hippoboscidae were updated, and four species were recognized within the *Crataerina* genus. All four are parasites of Apodiformes and Passeriformes, and are characterized by short and broad wings (Oboňa et al., 2022). *Crataerina acutipennis*, *C. melbae* and *C. pallida* have wings longer than the hind femur, while *C. obtusipennis* has wings shorter than the hind femur and broadly rounded wing tips (Oboňa et al., 2022)**.** Both sexes take blood meals and can be morphologically differentiated by the bigger abdomen of the females and the protrusion of the *aedeagus* in the males (Oboňa et al., 2022). The *Crataerina* genus is constituted by species considered to have relatively high host specificity (Lehikoinen et al., 2021). Particularly, *C. melbae* has shown a high specificity to Alpine swifts (*Tachymarptis melba*, syn. *Apus melba*) (Tella et al., 1998).

Swifts are an unusual group of birds, spending most of their lives in flight and landing only when breeding. Although this aerial lifestyle greatly reduces their likelihood of being stung by arthropods and infected by vector-borne parasites, swifts can still be heavily infested during breeding by permanent ectoparasites such as louse flies. Alpine swifts show high prevalence and intensity of infestation with *C. melbae* (Tella et al., 1998; Bize et al., 2003), that so far were reported to have only minor effects on the overall growth and weight of the nestlings, even at high burdens (Bize et al., 2003, 2004; Tella et al., 1995). Alpine swifts usually arrive in their breeding grounds in April and lay their eggs in mid-May. Nestlings hatch after 18–22 days of incubation in early June, with both parents feeding the offspring until fledging at around 50-70-days-of-age (Bize et al., 2004). Louse flies start emerging from pupae in late May, which coincides with the start of egg laying and hatching of the nestlings. *Crataerina* louse flies are larviparous/pupiparous, with the eggs and larvae developing inside the female. After laying, the larvae quickly pupate. The population of louse-flies builds up quickly until early July, before collapsing and generally producing only one generation per season (Bize et al., 2003, 2004; Tella et al., 1995; Tella and Jovani, 2000).

A study conducted on common swifts (*Apus apus*) and Alpine swifts from Italy and Switzerland did not detect haemosporidian parasites in the birds or in their associated louse flies (Ilahiane et al., 2023). In the same study, three haplotypes of the mitochondrial cytochrome-oxidase subunit I (*COI*) gene were identified in *C. melbae* infecting Alpine swifts in Switzerland (Ilahiane et al., 2023).

However, the intensive monitoring of the Swiss population of Alpine swift colonies has in recent years revealed a strong increase in nestling mortality associated with the presence of an avian *Trypanosoma* sp. (Cigler et al., 2024). The diseased Alpine swift nestlings show extensive subcutaneous haemorrhages, pale mucous membranes and primary feather damage associated with high *Trypanosoma* burdens in blood. Based on morphometric similarities to *T. bouffardi* described in songbirds in Africa, with no molecular data available (Molyneux, 1973), the species has been initially coined as *Trypanosoma bouffardi*-like (Cigler et al., 2024). Although this *Trypanosoma* sp. has been identified in several Alpine swift colonies in Switzerland, the role of *Crataerina* flies in the transmission of this blood parasites remains unclear.

Some avian trypanosomes use louse flies as vectors, particularly those belonging to the *Trypanosoma corvi*/*T. culicavium* genetic group (Santolíková et al., 2022). *Trypanosoma bouffardi*-like has been shown to be molecularly closely related to *T. corvi* and lineage B13, with only two single nucleotide polymorphisms (SNPs) detected in a fragment of the 18S RNA gene sequence (Cigler et al., 2024; Santolíková et al., 2022). *Trypanosoma corvi* is known to use *Ornithomyia avicularia* louse flies as vectors (Mungomba et al., 1989). Given the high burdens of *Crataerina* sp. louse flies found within urban Alpine swift colonies, we hypothesize that they are the competent vectors of *T. bouffardi*-like. Competent vector capacity requires the identification of actively multiplying trypanosomes within the arthropod (Mungomba et al., 1989; Santolíková et al., 2022).

Considering that trypanosomiasis is an emerging disease endangering the Swiss Alpine swift populations, we investigated the potential role of the louse flies as vectors, as well as the association of *Crataerina* wing morphotypes and *COI* haplotypes with their vector capacity.

## 2 Materials and methods

Adult louse flies and pupae were collected from six urban Alpine swift colonies located in six different cities (Baden, Biel, Burgdorf, Lenzburg, Seengen, and Solothurn) across Switzerland and processed as fixed samples or native specimens. The adult specimens were examined under a stereomicroscope (Stemi 508, Zeiss, Germany), photographed (Axiocam 208 color, Zeiss, Germany), and identified using morphological keys (Büttiker, 1994; Oboňa et al., 2022).

**Fixed samples**: Adult flies (n = 61) were collected between 2021 and 2023 from Alpine swift colonies in Baden, Biel, Burgdorf, Lenzburg, Seengen, and Solothurn, and fixed in 96 % ethanol. The flies were dissected, and the samples were processed by PCR only. To evaluate the infection status, intestines of 3–5 louse flies per colony were collected and processed as pools. In addition, to assess the potential presence of trypanosomes on the louse flies’ surface due to contamination with their own faecal material, abdominal surface swabs from 3 to 5 flies per colony were examined. One pool of intestines (n = 6) and surface swabs (n = 6) per colony was examined. Additionally, one ethanol-fixed specimen was processed by Scanning Electron Microscopy (SEM), essentially as previously described (Rubiola et al., 2024).

**Native specimens**: 158 adult flies and 30 pupae from Alpine swift colonies in Burgdorf, Biel and Solothurn were collected during the spring-summer seasons (March to August) from 2023 to 2025, either directly from the birds or the nests during monitoring and sampling performed by the Swiss Ornithological Institute and/or the Institute of Fish and Wildlife Health (see Ethics declarations). The adult flies were collected between May–June (n = 57) and July–August (n = 101), transported to the laboratory in modified plastic tubes to allow airflow, and dissected under a stereomicroscope within 48 h. To detect motile trypanosomes, the intestines and the heads were dissected and placed separately on a slide with phosphate buffered saline (PBS, PH:7.2) and observed under a microscope (Nikon Eclipse Ci with a calibrated Nikon Camera model DFK 23UP031. The material was thereafter recovered, placed in DNase free 1.5 ml microtubes, and stored at −20 °C for subsequent molecular studies.

Additionally, groups of five pupae per colony (n = 15) were washed by vortexing in 100 μl PBS to examine surface contamination with *Trypanosoma* sp. The PBS was recovered and used for DNA extraction. Finally, groups of 5 pupae from each of the three colonies were placed in a container covered with gaze and left at room temperature (22 °C) until the imagoes hatched. All 15 pupae hatched, and the imagoes were examined to rule out potential vertical transmission of trypanosomes. Pools of fly surface swabs, pupae surface wash, and imagoes, as well as individual abdomens or fly intestines, were processed by DNA extraction and PCR.

## 3 Molecular studies

Total DNA from all the different samples was extracted using the Quick-DNA™ Fecal/Soil Microbe MiniPrep Kit (Zymo Research, USA), according to manufacturer's instructions.

Subsequently, a PCR targeting the 18S rRNA gene-fragment of *Trypanosoma* spp. using the internal primers SSU561F and SSU561R described by Noyes et al. (1999) was performed as previously described (Cigler et al., 2024).

The barcoding of the flies was performed by PCR and sequencing of a fragment of the *COI* gene using the primers LEP-F1,5′-ATTCAACCAATCATAAAGATAT-3′ and LEP-R1, 5′-TAAACTTCTGGATGTCCAAAAA-3’ (Hebert et al., 2004). This PCR was conducted using QIAGEN Multiplex Mix 2 × in a final volume of 12.5 μl, 0.15 μl of each primer (100 μM) and 1.25 μl of each DNA sample. The following cycling program was used: 95 °C for 15min, 6 cycles (94 °C for 45sec, 45 °C for 45sec, 72 °C for 45sec); 35 cycles (94 °C for 45sec, 51 °C for 60sec, 72 °C for 45sec); and 72 °C for 5min on a Flexlid Mastercycler (Eppendorf, Hamburg, Germany). Each run contained a positive control (DNA of *Crataerina pallida* from common swift, *Apus apus*) and a non-template control (NTC).

The amplification products of both PCRs were analysed using the QIAxcel Connect Gel capillary gel electrophoresis system (QIAGEN, Germantown, MD) using the QIAxcel DNA screening cartridge (QIAGEN). Selected PCR amplicons were purified using a commercial kit (DNA Clean & Concentrator-5, Zymo Research, Irvine, USA) following manufacturer's instructions and submitted for Sanger sequencing to Microsynth, Balgach, Switzerland (https://srvweb.microsynth.ch/) with the two primers used for each amplification. Sequences were aligned and analysed using the Geneious Prime software (https://www.geneious.com). The resulting consensus sequences were compared with those available in GenBank by nucleotide BLAST analysis (http://blast.ncbi.nlm.nih.gov/Blast.cgi).

## 4 Statistical analysis

Variations in the frequency of *Trypanosoma* sp. detection in louse flies according to *Crataerina* wing morphotypes, *COI* haplotypes and sex were tested using the chi-square test in R cran (R Core Team, 2021).

## 5 Results

**Fig. 1:**
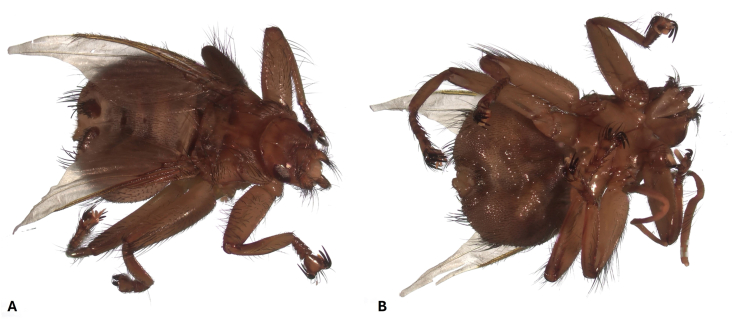
Dorsal (**A**) and ventral (**B**) view of a female *Crataerina melbae* louse fly under stereomicroscope.

**Fig. 2:**
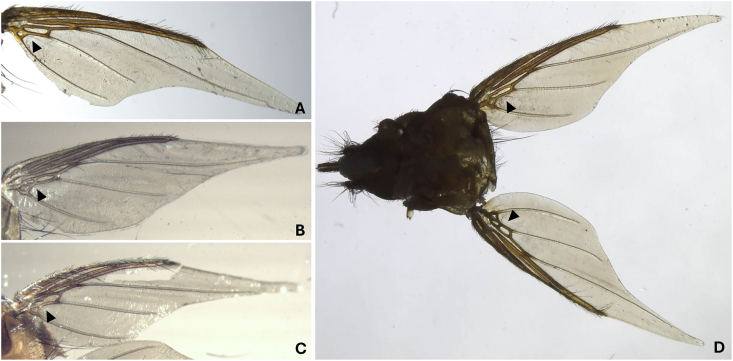
Wing morphology observed under stereomicroscope based on cell structure (arrowhead): *C. melbae* type (**A**), *C. acutipennis* type (**B**), intermediate type (**C**) and mix morphotype with each wing showing a different morphotype (**D**).

**Fig. 3:**
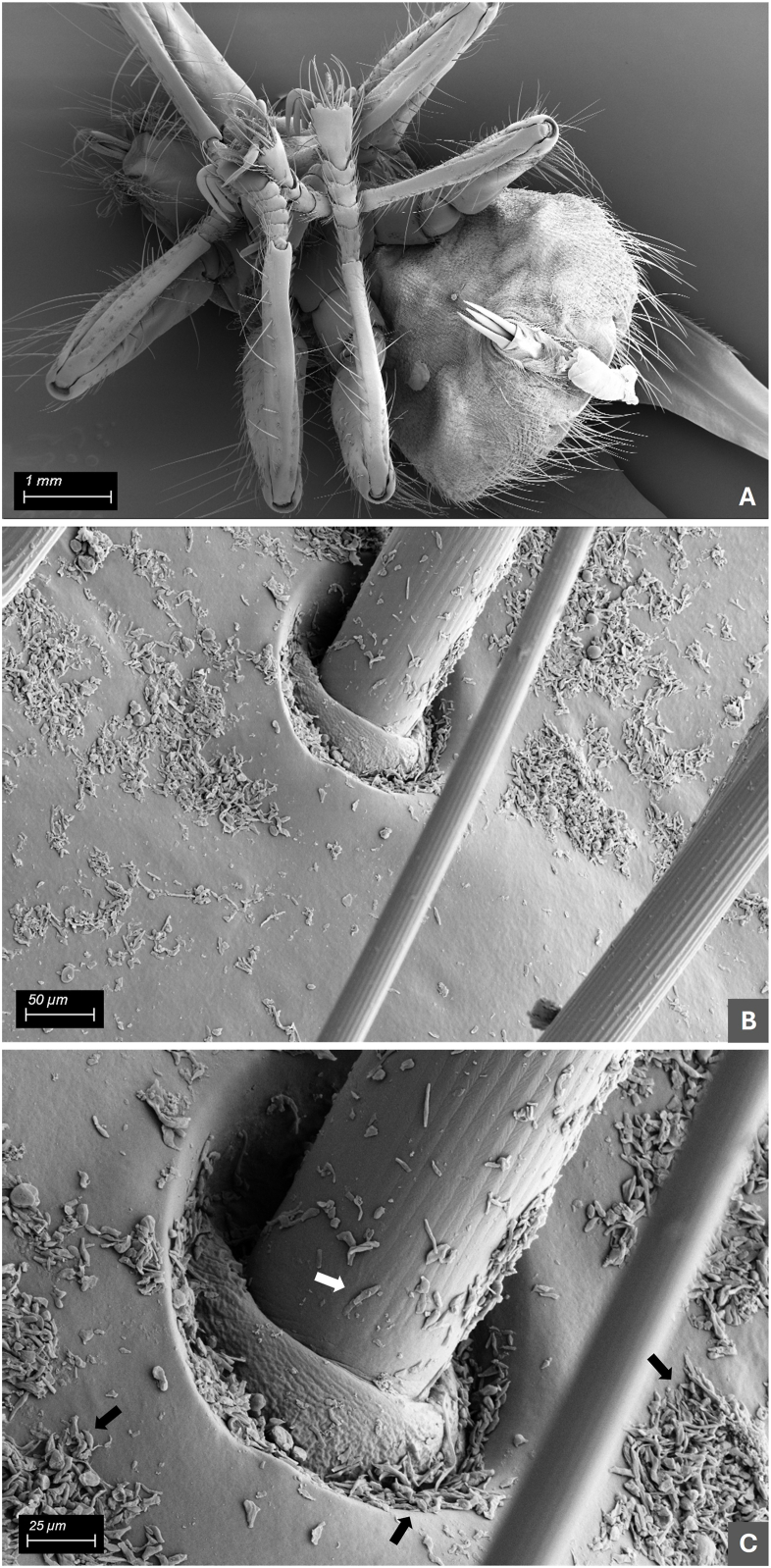
Scanning electron microscopy images of the ventral abdominal surface of a male *C. melbae* louse fly. At higher magnifications, fine, elongated material of about 10–15 μm covering the surface, presumed to be *Trypanosoma* sp., can be observed (arrows).

Morphologically, all 61 ethanol-fixed specimens were assigned to the genus *Crataerina* (Fig. 1). However, based on the determination key to Oboňa et al. (2022), which relies on the wing shape and venation, ethanol-fixed specimens were identified as *C. melbae* (n = 26) or *C. acutipennis* (n = 24) (Fig. 14 - *C. melbae* and 15 - *C. acutipennis* from Oboňa et al., 2022), whereas 11 specimens showed one wing of each type; i.e. an intermediate morphotype (Fig. 2). Hereafter, these are referred to as “mix”. All wing morphotypes were observed in all six colonies. Three of six ethanol-fixed louse fly intestinal pools from the colonies in Baden, Burgdorf and Solothurn, and three of six pooled abdominal surface swabs (two from *C. melbae* morphotype from Seengen and Solothurn, and one from *C. acutipennis* from Baden) resulted positive in the *Trypanosoma* spp. PCR. The consensus sequences obtained (n = 5, 539 bp, primers trimmed) were identical to the ones obtained from tissues of infected Alpine swifts (OR598759, Cigler et al., 2024). One representative *Trypanosoma* sequence from pooled abdominal surface swab (fixed material) was submitted to the GenBank (PX589732). Finally, one male specimen with the *C. acutipennis* wing type was processed by SEM and structures resembling trypanosomes were observed on the ventral abdominal surface (Fig. 3).

**Fig. 4:**
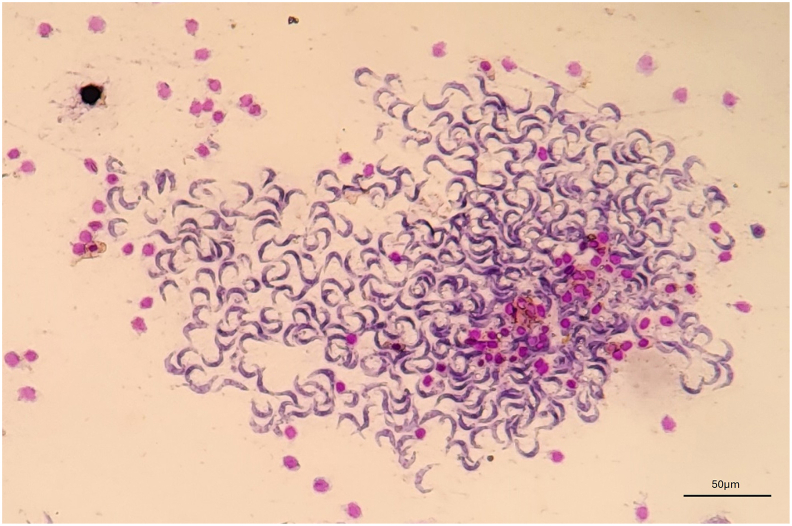
Diff-Quik® stained midgut content of a louse fly containing a large group of trypomastigotes. 200X

**Fig. 5:**
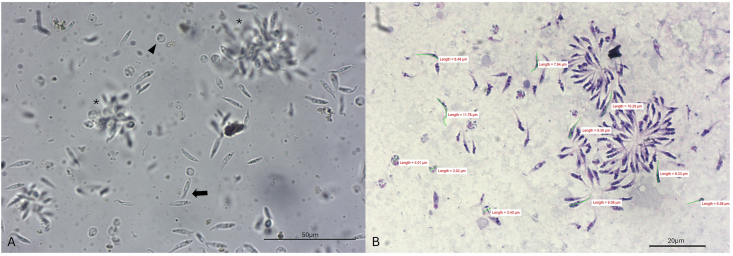
Unstained wet mount of rectal content of a louse fly (**A**) containing numerous pleomorphic epimastigotes (arrow) and choanomastigote-like forms (arrowhead). Groups of epimastigotes are visible in the background (asterisk). Diff-Quik® stained and Entellan® (Merck, Darmstadt, Germany)-mounted sample of the rectal content of the same louse fly (**B**) with measurements of pleomorphic trypanosomes. 400X

Microscopical inspection of 158 fresh louse-flies revealed that 21 (13.8 %) of them contained motile and actively multiplying trypanosomes in the midgut (Fig. 4) and hindgut, with particularly high amounts of pleomorphic epimastigotes and trypomastigotes in rectum, alongside fewer choanomastigote-like forms (Kaufer et al., 2017) (Fig. 5, Suppl. File 1). All the *Trypanosoma*-positive flies were collected between July and August (20.8 %, 21/101). Trypanosomes were not detected on the head portion of the louse flies. Using the determination key described above, these 158 specimens were identified as *C. melbae* (n = 86), *C. acutipennis* (n = 37) and mix wing morphotypes (n = 35). The frequency of *Trypanosoma* detection in the specimens collected in July–August (n = 101, 64 females and 37 males) significantly differed between male (13/37; 35.1 %) and female louse-flies (8/64, 12.5 %) (chi-square test: *Χ*^2^ = 7.27, *P* = 0.007). However, trypanosomes were less frequently detected in *C. melbae* morphotype (8/86, 9.3 %) compared to the mix (7/35, 20 %) and *C. acutipennis* (6/37, 16.2 %) morphotypes (*Χ*^2^ = 34.9, *P* < 0.001). The three pools of pupae surface wash and of *in vitro*-hatched imagoes were negative by *Trypanosoma* sp. PCR. All the specimens that had tested positive for trypanosomes by microscopy also resulted positive by *Trypanosoma* spp. PCR. Six PCR products were sequenced. All consensus sequences (539 bp, primers trimmed) were identical amongst each other and with the sequence reported as *Trypanosoma* sp. from Alpine swifts (OR598759). One representative sequence from a sample containing motile and multiplying trypanosomes was submitted to the GenBank (PX589733).

*COI*-based barcoding resulted in 24 consensus sequences (665 bp; primers trimmed) representing six different haplotypes, with identities ranging between 100 and 99.4 % (up to 4 SNPs) amongst them. Four of these haplotypes were detected in several louse flies each (7, 5, 5 and 5, respectively) while the remaining two were each found in a single louse fly. The four most frequent haplotypes were detected in flies with all three different wing morphotypes, and in all three colonies. Five specimens from one single nest in one colony (Solothurn) resulted in five different haplotype sequences.

**Fig. 6:**
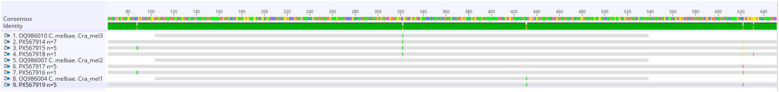
Multi-alignment of the six representative cytochrome-oxidase 1 (*COI*) gene sequences of *Crataerina melbae*, together with the representative sequences of Haplotypes Cra_mel1, 2 and 3 (Geneious prime software). Noteworthy are the five positions where variability is detectable (from 1 to 4 SNPs).

**Fig. 7:**
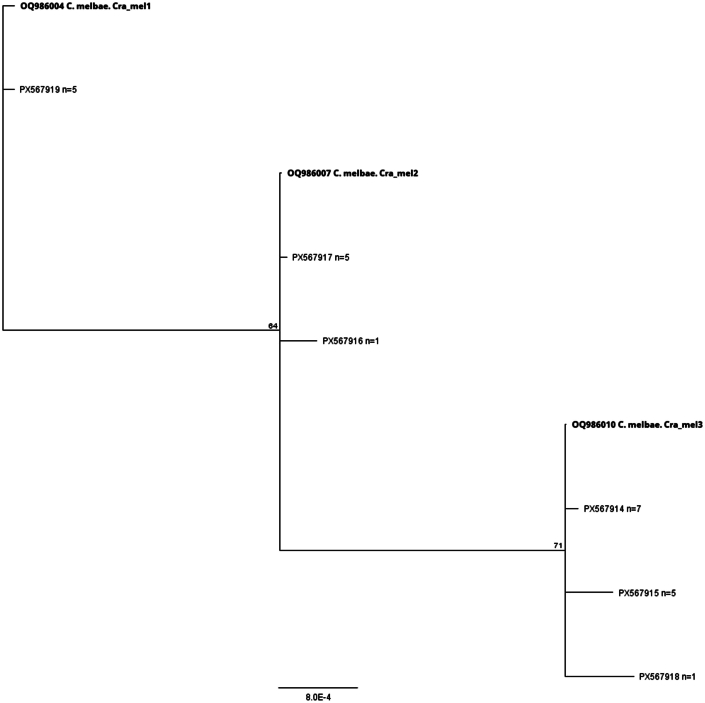
Phylogenetic tree resulting from the multi-alignment of cytochrome-oxidase 1 (*COI*) gene sequences shown in Fig. 6. A Neighbor-Joining method and no outgroup was used (Geneious prime software). The obtained sequences are grouped together with the representative Cra_mel1 (n = 1), 2 (n = 2) and 3 (n = 3), with a moderate support.

In detail, the sequences showed a 100 % coverage and an identity of 92.8–92.9 % with *Crataerina pallida* (OX453295) and 92.8–93.1 % with *Ornithomya fringillina* (OZ021696). All sequences showed a 100 % identity with 65 % coverage with *C. melbae* sequences as follows: 13 with *C. melbae* from Biel (OQ986010-13) previously identified as **Haplotype Cra_mel3**; six with *C. melbae* from Solothurn (OQ986007-9), previously identified as **Haplotype Cra_mel2** and five with 100 % identity with *C. melbae* from Biel and Solothurn (OQ986004-6) previously identified as **Haplotype Cra_mel1** (Ilahiane et al., 2023). One representative sequence for each group as well as the two unique sequences were submitted to the GenBank (PX567914-PX567919). The alignment of these six sequences (with frequency of each sequence type), together with representative sequences of Haplotypes Cra_mel1, 2 and 3, as well as a phylogenetic tree constructed with the Neighbor-Joining method and no outgroup (Geneious prime software) are shown in Fig. 6, Fig. 7, respectively.

Fly specimens showing all four most frequent *COI* haplotypes, as well as one of the unique *COI* sequences harboured active and multiplying trypanosomes.

Despite the SNPs observed among the six different *COI* haplotypes, all the translated proteins (n = 24) were 100 % identical. Following a BLASTp, the identity was 97.7 % with *COI* protein sequences from louse flies (*C. pallida*, *O. fringillina*, and *O. avicularia* among others).

## 6 Discussion

In recent years, avian trypanosomiasis caused by *T. bouffardi*-like has been pinpointed as a potential threat to the Swiss population of Alpine swift (Cigler et al., 2024). Since all monitored urban Alpine swift colonies in Switzerland also harbour *C. melbae* (Bize et al., 2003, 2004; Ilahiane et al., 2023), the potential role of *C. melbae* in the transmission of *T. bouffardi*-like was investigated. Initially, ethanol fixed louse fly specimens collected during episodes of mass nestling mortalities attributed to *T. bouffardi*-like infection were analysed. The detection of *T. bouffardi*-like DNA in the intestines of the louse flies could have been attributed to the sole “passage” of parasites after a blood meal from an infected bird (Santolíková et al., 2022). However, the observation of *Trypanosoma-*like structures on the ventral abdominal surface of a louse fly by SEM, and the PCR positive surface swabs, suggested active multiplication of the protozoans within the arthropods. This hypothesis was confirmed by the detection of actively multiplying trypanosomes in the midgut and rectum of the fresh specimens (**Suppl. File 1**). Interestingly, only *Crataerina* adults collected in summer (July–August) were confirmed as infected with *Trypanosoma* sp., while the adults collected from birds and nests soon after pupal hatching in May and June, as well as pupae and *in vitro*-hatched imagoes, were all negative, both by microscopy and PCR. Both males and females collected in July–August harboured multiplying trypanosomes, with a higher frequency in males (35.1 %) than females (12.5 %). These observations suggest that the flies become infected in early summer, amplify the trypanosomes, and serve as vectors for the nestlings. Given that the earliest-emerging louse flies during the nesting season are predominantly male and feed preferentially on feathered adult Alpine swifts (Bize et al., 2003, 2004; Tella and Jovani, 2000), it is suspected that the adult birds introduce the *Trypanosoma* into the colonies. Further male louse flies are presumed to act as the initial amplifiers of *Trypanosoma*, although this remains to be verified in detail.

Transmission routes have not yet been confirmed but may occur either by ingestion of the infected flies, or by cutaneous microlesions contaminated with faecal material of flies containing trypanosomes. Proper confirmation of transmission routes would require animal experimentation which exceeds the scope of this study. However, the high *Trypanosoma* epimastigote and trypomastigote concentrations found in the rectum of the examined flies (**Suppl. File 1**) are comparable to those described for *Trypanosoma corvi* in *O. avicularia* (Mungomba et al., 1989). It is possible that these two genetically related *Trypanosoma* species are frequently transmitted by contact with fly faecal material. The importance of the observed choanomastigote-like forms remains unclear (Kaufer et al., 2017). They may be a part of natural *Trypanosoma* life cycles, an environmentally hardy form ready for excretion, or dying parasites (Mungomba et al., 1989). Additionally, the effects of the *Trypanosoma* sp. infection on the behaviour of louse flies warrants further research, as feeding and defecating habits may be influenced by infection. For example, some *Trypanosoma cruzi*-infected triatomine kissing bugs (*Triatoma infestans*) have been shown to change their behaviour by ingesting larger blood volumes and defecating more frequently (Verly et al., 2020).

The prevalence of infected louse flies in the present study (13.3 %) was lower than that observed in *O. avicularia* with *T. corvi* (42 %) performing a similar microscopic approach (Mungomba et al., 1989). Nevertheless, if only the flies obtained in July–August are considered, the prevalence at this time point is slightly higher (20.8 %).

Since avian trypanosomiasis in the Swiss Alpine swift population appears to have emerged only in recent years, we attempted to identify whether the different *Crataerina* wing morpho- and/or *COI* haplotypes potentially differ in their vector capacity, which could explain the novel expansion of the disease. Through microscopic examination, different morphotypes of *Crataerina* were identified, coexisting in the same colonies, and even on the same birds or nests. All morphotypes were proven to be competent vectors. Despite this, a significantly higher proportion of *Trypanosoma* sp. positivity was detected in the mix and *C. acutipennis* morphotypes in comparison to *C. melbae*. Whether the morphotype could be associated with different feeding behaviours, or digestive processes allowing for increased trypanosome infection rates and/or trypanosome multiplication, requires further investigation.

Additionally, our findings suggest that the previously morphologically described species *C. melbae* and *C. acutipennis* (Oboňa et al., 2022), represent minimal morphological variations from a single species sharing both host and microhabitat, i.e. Alpine swift nestlings and adults. This is further supported by the presence of mixed morphotypes, which *COI-*based barcoding unanimously identified as *C. melbae* (regardless of the different morphotypes). This barcoding confirmed the presence of the three previously defined haplotypes of *C. melbae* (haplotypes Cra_mel 1 to 3) originally identified in flies collected from the same urban Alpine swift colonies (Ilahiane et al., 2023). However, since the sequences generated in this study were ∼35 % longer than those previously reported (OQ986004-OQ986013; Ilahiane et al., 2023), we were able to detect additional variation outside the previously described fragment, and six different sequence types were detected (Fig. 6). Within the sequences assigned to haplotypes Cra_mel 3 and Cra_mel 2 (Ilahiane et al., 2023), we identified further SNPs beyond the core region and observed three variants (two major ones building two subgroups of seven and five sequences each, and one unique sequence), and two sequence variants (one variant repeated 5 times and one unique sequence), respectively. All *COI* barcode haplotypes were identified in specimens acting as vectors. Moreover, five specimens were identified within a single nest, each with a different sequence type, and all were infected with multiplying trypanosomes.

Interestingly, the differences observed among sequences (up to four SNPs) do not affect the translated protein and are therefore silent mutations. This was not analysed in the original description of the barcoding using *COI* DNA sequences (Hebert et al., 2004), nor in other similar studies performed in louse flies (Ilahiane et al., 2023; Lehikoinen et al., 2021). Translation and protein analysis is important to properly define the inter- and intraspecies variability. This study shows that all analysed specimen sequences (n = 24) produced the same protein fragment, therefore reinforcing the presence of a single species, namely *C. melbae*, infecting Alpine swifts (Lehikoinen et al., 2021).

The identity and specificity of the *Trypanosoma* sp. affecting Alpine swifts remain uncertain. These trypanosomes have been coined as *T. bouffardi*-like based on the morphological similarities with the species described in songbirds in Africa (Cigler et al., 2024; Molyneux, 1973). However, as no sequence reports or stored genetic material are available for *T. bouffardi* (Molyneux, personal communication) proper comparisons are not feasible at this time. The vector competency of *C. melbae* for *T. bouffardi*-like, and their specificity to Alpine swifts, may suggest a long-term host-parasite-vector relationship. This, however, does not align with the only recently observed increase in nestling mortalities (Cigler et al., 2024). As such, whether this is a long-term host-parasite relation, or a truly recent adaptation, remains to be confirmed. Whether this cycle is also happening in more natural, non-monitored cliffside breeding colonies, is unknown. Likewise, it is unknown whether other birds in Switzerland and Europe are also infected with *T. bouffardi*-like, especially considering that most wild bird species are not monitored as closely as the Alpine swifts. Moreover, the potential initial spillover from other bird species to the Alpine swifts by ingestion, or even bites, from trypanosome-infected insect vectors cannot be completely ruled out. The wide migratory range of the Alpine swifts further expands the potential sources of infection.

In conclusion, this study suggests that the louse-flies *C. melbae* and *C. acutipennis* may constitute a single species (i.e. *C. melbae*) which, regardless of the morphotypes of their wings or their *COI* haplotypes, can act as competent vector for *Trypanosoma* sp. parasites affecting Alpine swifts. Future studies should be focused on environmentally sustainable ways to control *C. melbae* to minimize transmission and the impact of trypanosomiasis in Alpine swift populations.

## CRediT authorship contribution statement

**Gastón Moré:** Writing – review & editing, Writing – original draft, Visualization, Methodology, Investigation, Formal analysis, Conceptualization. **Pia Cigler:** Writing – review & editing, Writing – original draft, Visualization, Methodology, Investigation, Formal analysis, Data curation, Conceptualization. **Pierre Bize:** Writing – review & editing, Resources, Data curation. **Andrew Hemphill:** Writing – review & editing, Software, Resources. **Saskia A. Keller:** Writing – review & editing, Resources, Funding acquisition. **Caroline F. Frey:** Writing – review & editing, Supervision. **Walter Basso:** Writing – review & editing, Supervision, Conceptualization.

## Ethics declarations

Monitoring of Alpine swift colonies was performed by the Swiss Ornithological Institute in accordance with national legislation following National Animal Experimentation Permit Numbers 34497 and 37842, as well as from the Fish and Wildlife Health Institute, Vestuisse Faculty, Bern University under the National Animal Experimentation Permit Number 34943.

## Funding

This work was partially financed by Swisslos Solothurn [2023/1202]; and Swisslos Aargau [2923.REF 5933].

## Declaration of competing interest

All the authors are free from conflict of interests which could potentially bias the present study.

## Appendix A Supplementary data

Supplementary File 1 Video of the wet mount of rectal content of a louse fly from Fig. 5, containing numerous active pleomorphic epimastigotes in groups and some choanomastigote-like forms in the background. The video was taken with a Samsung S10E mobile phone. Supplementary File 1
